# *In utero* hematopoietic cell transplantation: induction of donor specific immune tolerance and postnatal transplants

**DOI:** 10.3389/fphar.2014.00251

**Published:** 2014-11-12

**Authors:** William H. Peranteau

**Affiliations:** Department of Surgery, Center for Fetal Research, The Children’s Hospital of PhiladelphiaPhiladelphia, PA, USA

**Keywords:** *in utero*, immune tolerance, postnatal transplant, fetus, hematopoietic stem cell, myeloablation, immunosuppression, *in utero* hematopoietic cell transplantation

## Abstract

*In utero* hematopoietic cell transplantation (IUHCT) is a non-myeloablative non-immunosuppressive transplant approach that allows for donor cell engraftment across immunologic barriers. Successful engraftment is associated with donor-specific tolerance. IUHCT has the potential to treat a large number of congenital hematologic, immunologic, and genetic diseases either by achieving high enough engraftment levels following a single IUHCT or by inducing donor specific tolerance to allow for non-toxic same-donor postnatal transplants. This review evaluates donor specific tolerance induction achieved by IUHCT. Specifically it addresses the need to achieve threshold levels of donor cell engraftment following IUHCT to consistently obtain immunologic tolerance. The mechanisms of tolerance induction including partial deletion of donor reactive host T cells by direct and indirect antigen presentation and the role of regulatory T cells in maintaining tolerance are reviewed. Finally, this review highlights the promising clinical potential of *in utero* tolerance induction to provide a platform on which postnatal cellular and organ transplants can be performed without myeloablative or immunosuppressive conditioning.

## INTRODUCTION

The fetal environment offers the unique opportunity to take advantage of the developing immune system to induce immunologic tolerance to foreign antigen. This was initially recognized in an experiment of nature in which Owen observed permanent red blood cell chimerism in dizygotic cattle twins that shared cross-placental circulation (Owen, 1945). Later studies by Billingham, Medawar, and others confirmed the ability to induce immunologic tolerance by early gestational exposure to foreign antigen (Anderson et al., 1951; Billingham et al., 1952; Simonsen, 1955). *In utero* hematopoietic cell transplantation (IUHCT) seeks to take advantage of this developmental phenomenon. In multiple animal models, IUHCT has been shown to be a non-myeloablative non-immunosuppressive transplant approach that allows for engraftment across immunologic barriers and is associated with the induction of donor specific tolerance (Flake and Zanjani, 1999; Kim et al., 1999; Peranteau et al., 2002). Clinically, IUHCT has the potential to treat any congenital hematologic, genetic or immunologic disorder which can be prenatally diagnosed and which is currently managed with a postnatal hematopoietic stem cell (HSC) transplantation requiring a matching donor and/or myeloabalative and immunosuppressive conditioning.

**FIGURE 1:**
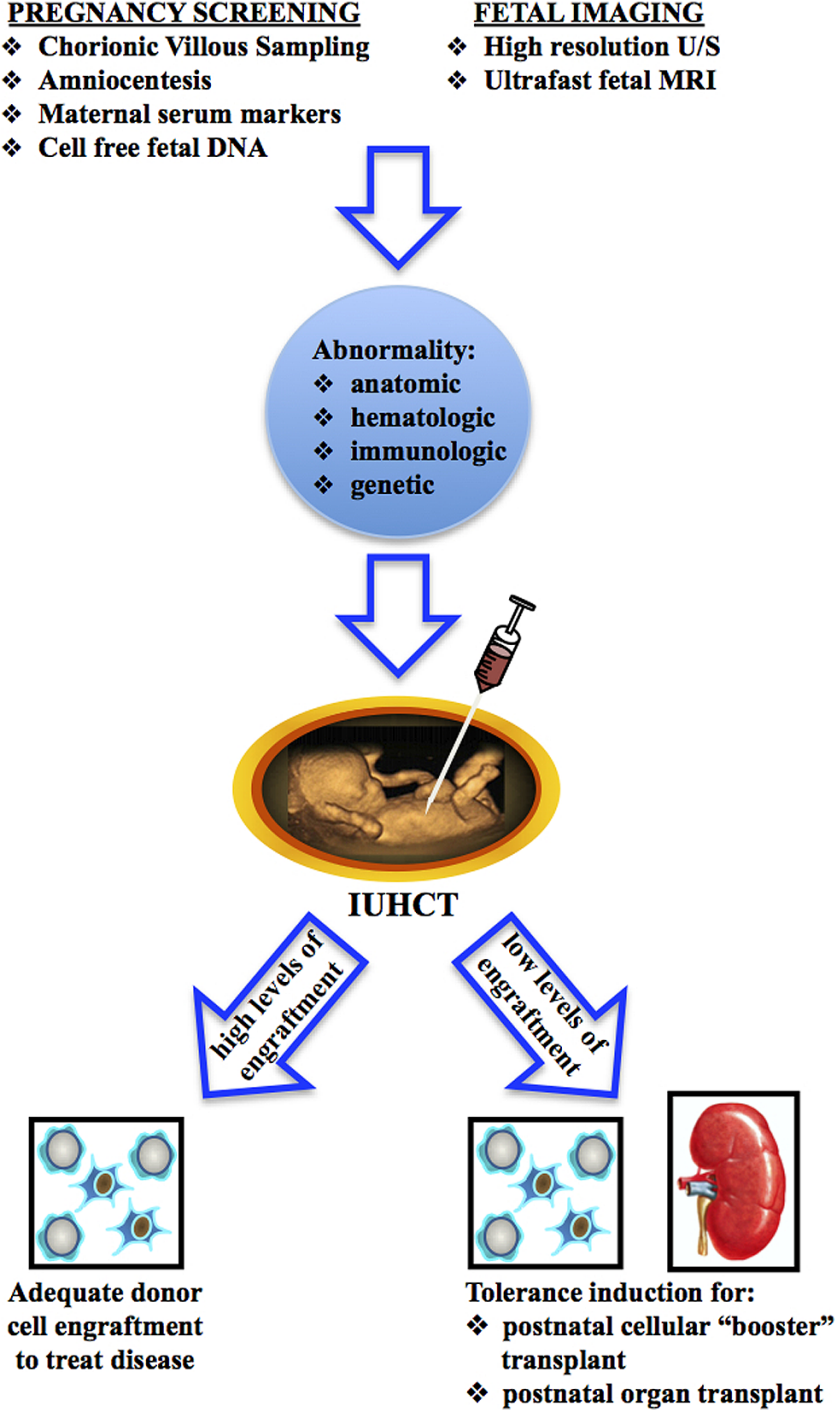
**Two approaches for the clinical application of IUHCT**.

The clinical application of IUHCT could take one of two potential courses (**Figure 1**). A single *in utero* transplant may result in high enough levels of donor cell engraftment to treat the target disease. Alternatively, IUHCT may be used to induce donor specific tolerance which would allow for postnatal same-donor transplants with non-toxic conditioning regimens to increase donor cell engraft to clinically relevant levels. Tolerance achieved by IUHCT may also be used to permit postnatal same-donor organ transplants without immunosuppressive conditioning. To date, IUHCT has only been clinically successful in the treatment of severe combined immunodeficiency disorder (SCID; Flake et al., 1996; Wengler et al., 1996). Broader clinical application of IUHCT is limited by the ability to consistently achieve high enough levels of donor cell engraftment to treat the target disease. Thus, tolerance induction by IUHCT to allow for postnatal “booster” transplants may be instrumental to the future clinical application of IUHCT. In this review, we focus on the progress that has been made in understanding and achieving immunologic tolerance following IUHCT and how this tolerance can be used as a platform for non-myeloablative non-immunosuppressive postnatal transplants to either achieve clinically acceptable levels of engraftment or allow for solid organ transplants.

## IUHCT AND ALLOGENEIC ENGRAFTMENT: FROM MICRO TO MACROCHIMERISM AND TOLERANCE INDUCTION

*In utero* hematopoietic cell transplantation has been studied in multiple animal models. Initial results in the sheep model were very encouraging demonstrating stable long-term hematopoietic chimerism in three of four sheep following IUHCT (Flake et al., 1986). Unfortunately, these findings did not translate into similar results in clinical studies. Successful engraftment following IUHCT in humans has been limited to circumstances of immunodeficiency and those in which a donor cell selective advantage exists (Flake et al., 1996; Wengler et al., 1996; Gil et al., 1999; Bartolome et al., 2002; Pirovano et al., 2004; Touraine et al., 2004; Muench, 2005; De Santis et al., 2011). These discouraging results highlighted the need for a more in depth study of the events following IUHCT including the induction of donor specific tolerance. To this aim, murine models of IUHCT have been developed. Studies in these models support an intimate relationship between the levels of donor cell chimerism following IUHCT and tolerance induction. In chimeric mice in which donor cell engraftment was only detectable by PCR and undetectable by flow cytometry (microchimerism), donor specific tolerance, as demonstrated by skin graft acceptance, response to postnatal boosting transplants, and *in vitro* proliferation assays, is inconsistent and occurs in only a subset of animals (Billingham et al., 1953; Carrier et al., 1995; Kim et al., 1999). Interestingly, studies in mice and large animals have found that tolerance following IUHCT may persistent even when peripheral blood chimerism levels are low if donor cells persist in tissues or the peritoneal cavity of recipients (Carrier et al., 1995; Mathes et al., 2001, 2005; Chen et al., 2004). Technical advances, including the ability to deliver higher doses of donor cells at the time of IUHCT via an intravenous injection, have allowed for the creation of mice with chimerism levels consistently greater than 1% (macrochimerism; Peranteau et al., 2006, 2007). The ability to achieve higher initial levels of donor cell engraftment has demonstrated that induction of donor specific tolerance can be consistently achieved in macrochimeric animals and tolerance correlates with donor chimerism levels (Hayashi et al., 2002; Ashizuka et al., 2006). Specifically, 60% of mice with peripheral blood chimerism levels of less than 1%, and 100% of mice with chimerism levels greater than 1% following IUHCT demonstrated successful enhancement of allogeneic engraftment following postnatal, same-donor, bone marrow (BM) transplants suggestive of the presence of donor specific tolerance. Decreased donor specific reactivity was demonstrated by MLR in those mice with <1% chimerism in which engraftment could be successfully enhanced following IUHCT compared to those in which engraftment could not be enhanced (Ashizuka et al., 2006). In another study, peripheral blood chimerism was noted to correlate with thymic chimerism and donor specific tolerance as measured by skin graft acceptance. In this study, chimerism levels greater than 3% at the time of skin graft placement were consistently associated with donor specific tolerance and graft acceptance (Chen et al., 2010). In this study, adequate levels of donor cell engraftment were needed for the induction of tolerance. However, peripheral blood chimerism was not required for the maintenance of tolerance as demonstrated by persistence of donor skin grafts despite the loss of peripheral blood chimerism in some mice.

## IUHCT AND MECHANISM OF DONOR SPECIFIC TOLERANCE

Fetal immunologic tolerance is a phenomenon believed to be temporally related to thymic development (Billingham et al., 1953). The developing fetal thymic microenvironment plays a primary role in the positive and negative selection of pre-T cells resulting in the deletion of presumed auto-reactive T cell clones with a high affinity for self antigen in association with self MHC while maintaining a T cell repertoire for foreign antigen (Sprent, 1995; Goodnow, 1996; Goodnow et al., 2005). In the human fetus, TCR bearing, single positive lymphocytes can be identified as early as 13–14 weeks gestation. In the murine system, this stage of development corresponds to ∼17 days gestation. Thus, IUHCT attempts to introduce donor cells into the fetal thymic microenvironment prior to this time such that donor cells will be identified as “self” and donor antigens will undergo appropriate thymic antigen presentation resulting in clonal deletion of donor alloreactive host T cells.

Although donor specific tolerance following IUHCT is well accepted, the mechanisms underlying this tolerance have only recently begun to be understood. Early studies suggested tolerance was the result of partial deletion of donor specific host T cells combined with peripheral suppression of donor reactive T cells that escape deletion (Kim et al., 1999; Nijagal et al., 2011). Thymic deletion of donor reactive host T cells can occur via the direct pathway in which donor antigen is presented by donor antigen presenting cells (APCs) or the indirect pathway in which recipient APCs process donor derived allo-MHC molecules into peptides and then present those peptides to T-cells on self-class II MHC molecules. Additionally, the “semidirect” pathway whereby intact donor MHC molecule-donor peptide complexes are taken up by host APCs and directly interact with reactive T cells may be involved (Herrera et al., 2004; Nijagal et al., 2013). Initial studies using the mammary tumor virus (*Mtv*) superantigen system demonstrated that partial deletion of donor reactive host lymphocytes occurs via both the indirect and direct route of antigen presentation following IUHCT (Shaaban et al., 2000; Peranteau et al., 2002). More recently, murine studies using TCR-transgenic systems that allow differentiation of direct vs. indirect antigen presentation with subsequent donor reactive T cell deletion confirm that deletional tolerance can occur via both pathways (Nijagal et al., 2013). In this study, expression of donor-derived class II antigens on host APCs was assessed to determine the possible contribution of the “semidirect” pathway to deletion of donor reactive T cells. No expression was seen suggesting that the “semidirect” pathway does not play a significant role in deletional tolerance following IUHCT. In addition to inducing immunologic tolerance of host cells to donor cells, IUHCT also results in partial deletion of host reactive donor T cells derived from hematopoietic stem or early progenitor cells at the time of IUHCT via the direct pathway (Bacchetta et al., 1993; Shaaban et al., 2000; Peranteau et al., 2002). In these studies, the direct route of antigen presentation was more efficient with respect to the degree of relevant clonal deletion, but neither route resulted in complete deletion of donor (or host) reactive lymphocytes. Remaining donor (or host) reactive lymphocytes are thought to be suppressed in the periphery by mechanisms that remain to be fully elucidated. This is similar to clinical experience in children who have undergone a successful IUHCT for SCID. These children were immunologically tolerant and were shown to have residual clones of donor reactive cells that were anergic in proliferative assays (Roncarolo et al., 1988; Sakaguchi et al., 1995; Touraine et al., 2005). The mechanism by which this occurs is hypothesized to be related to peripheral regulatory cells using the natural mechanisms of controlling autoreactive T cells that escape thymic deletion (Muench, 2005). In the murine model, the contribution of CD4^+^CD25^+^Foxp3^+^ T regulatory cells to this process remains unclear. Studies have shown an increase in the percentage of Treg cells (as well as the Treg/Teff ratio) in the thymus and spleen of chimeric mice following IUHCT related to deletion of the Teff population but not an increase in the absolute number of Treg cells (Nijagal et al., 2013). Although this shift in the Treg/Teff ratio may play an important role in the establishment of engraftment, the contribution of Tregs to maintaining chimerism following IUHCT remains to be shown.

## *IN UTERO* TOLERANCE INDUCTION AND POSTNATAL TRANSPLANTS

Technical improvements in injection techniques have highlighted the intravascular route as a promising alternative to the intraperitoneal route of injection. IUHCT via the intravascular route has achieved initial chimerism levels of 1–23 and 3–39% in the murine model and the preclinical canine model respectively (Peranteau et al., 2006; Vrecenak et al., 2014). These results are encouraging and those animals at the higher end of the engraftment spectrum have donor cell chimerism levels that may be adequate to treat the target disease. However, studies in murine models of Sickle cell anemia, a primary target disease for treatment by IUHCT, suggest that 70 and 40% donor cell myeloid engraftment is needed to eliminate peripheral RBC sickling and anemia respectively (Iannone et al., 2001). To obtain these and higher levels of engraftment in all recipients of IUHCT, alternative approaches must be explored. *Ex vivo* modification of donor HSCs or *in vivo* treatment of fetal recipients with agents which provide a competitive advantage to donor HSCs over endogenous fetal HSCs may be employed to increase donor cell engraftment to clinically relevant levels following a single IUHCT (Peranteau et al., 2006; Derderian et al., 2014). Alternatively, donor specific tolerance induction by IUHCT can be used as a platform on which postnatal transplants using the same prenatal donor source can be performed following non-myeloablative, non-immunosuppressive conditioning to increase engraftment levels.

A review of the literature reveals multiple studies demonstrating the feasibility of tolerance induction by IUHCT followed by postnatal same-donor “booster” transplants (**Table 1**). In the murine model, allogeneic donor cell engraftment was minimally increased when postnatal same-donor transplants were performed in the absence of any conditioning regimen (Carrier et al., 1995; Donahue et al., 2001). We performed additional studies in which non-myeloablative non-toxic conditioning regimens, including low dose total body irradiation (TBI) or busulfan, were administered to chimeric recipients prior to a postnatal same-donor transplant (Peranteau et al., 2002; Ashizuka et al., 2006). Engraftment enhancement directly correlated with the dose of TBI or busulfan administered with near complete donor cell chimerism achieved at the highest doses. The increase in donor cell chimerism resulted from the postnatal donor cell source as opposed to expansion of donor HSCs which had engrafted following IUHCT. Finally, chimeric mice in which engraftment was successfully enhanced demonstrated reduced donor cell reactivity of recipient cells by MLR following IUHCT and prior to the postnatal transplant. These studies highlight the potential to increase allogeneic donor cell engraftment to clinically relevant levels by a combination of tolerance induction by IUHCT and engraftment enhancement by a postnatal BMT using the same prenatal donor. They demonstrate the need for some conditioning regimen to provide a competitive advantage to the donor cell population to achieve the desired engraftment levels independent of preexisting immunologic tolerance. Studies in the preclinical canine model also support the feasibility of this approach with results that reflect similar findings to those achieved in the murine model. Specifically, we demonstrated the ability to successfully enhance peripheral blood donor cell chimerism in the canine model by a combination of IUHCT and a postnatal same-donor BMT using a low-dose busulfan conditioning regimen (Peranteau et al., 2009). In this study, donor chimerism levels were increased from <1 to 35–50% and remained stable up to 6 months to 1 year after transplant in two of six recipients. Control dogs which did not receive an IUHCT never demonstrated any donor cell engraftment following postnatal BMT. The 33% success rate of enhancing engraftment in dogs with initial chimerism levels <1% following IUHCT concurs with murine studies in which 60% of mice with chimerism levels <1% following IUHCT successfully enhanced donor cell engraftment using a similar postnatal transplant regimen (Ashizuka et al., 2006). In both studies, failure to achieve stable enhanced donor cell engraftment was associated with increased donor cell reactivity of recipient cells on MLR suggesting a lack of definitive tolerance. These studies support the need to achieve initial levels of donor cell engraftment >1% following IUHCT to reliably induce donor specific tolerance for postnatal cellular transplants. More recently, IUHCT via the intracardiac route in the canine model has more consistently resulted in donor cell engraftment at levels believed to be associated with donor specific tolerance (Vrecenak et al., 2014). These results highlight the potential to more reliably enhance donor cell chimerism by the combination of IUHCT and postnatal same-donor transplants in the clinical setting.

**Table 1:** Summary of studies using IUHCT to induce donor specific tolerance for postnatal allogeneic cellular or organ transplants.

Animal model	Postnatal transplant	Result	Study
Murine	BM (cellular)	Small increase in donor engraftment following unconditioned postnatal transplant (0.2–5% donor chimerism)	Carrier et al. (1995)
		Small increase in donor engraftment following unconditioned postnatal transplant (0.05–0.58 to 2.53%)	Donahue et al. (2001)
		Conversion to >90% donor cell engraftment following low dose non-myeloablative TBI and postnatal transplant	Peranteau et al. (2002)
		Conversion to near total donor cell chimerism following minimally myeloablative conditioning and postnatal transplant	Ashizuka et al. (2006)
Canine	BM (cellular)	Transient elevation of donor cell engraftment (<1–40% donor cell chimerism) in all recipients of an IUHCT and postnatal BM transplant following low-dose Busulfan conditioning. Sustained long-term enhancement of engraftment (donor cell chimerism: 35–50%) in two of six recipients	Peranteau et al. (2009)
Non-human primate	BM (cellular)	Persistent hyporesponsiveness to donor cells on mixed lymphocyte reaction but no significant increase in donor cell engraftment	Shields et al. (2004)
Murine	Skin graft	Prolonged skin graft acceptance in microchimeric mice	Carrier et al. (1995)
		Skin graft acceptance in 66% of microchimeric mice	Kim et al. (1998)
		Skin graft acceptance in 100% of macrochimeric mice	Hayashi et al. (2002, 2004)
		Donor cell chimerism levels >3% required to consistently accept postnatal skin grafts	Chen et al. (2010)
Ovine	Renal	Donor kidney rejected 10 days after transplant in sheep that had 3–5% donor cell engraftment following IUHCT	Hedrick et al. (1994)
Swine	Renal	Prolonged donor kidney survival after minimal immunosuppression for minor histocompatibility antigens	Mathes et al. (2001)
		Prolonged donor kidney survival with minimal or no immunosuppression and no evidence of anti-donor antibodies	Mathes et al. (2005)
		Renal allograft survival for >100 days without immunosuppression	Lee et al. (2005)
Canine	Renal	Long-term acceptance of donor kidney transplant without immunosuppression in four recipients; No evidence of rejection in three of four recipients (12–55% donor cell chimerism at transplant); Mild chronic rejection noted in recipient who had lowest donor cell chimerism (7%) at the time of transplant	Vrecenak et al. (2014)
Non-human primate	Renal	Prolonged survival of paternal kidney transplant in chimeric recipients (donor chimerism level <0.1%) of a paternal IUHCT vs. controls which did not receive an IUHCT (time to rejection: 1 vs. 4–7 weeks)	Mychaliska et al. (1997)

*All studies evaluated the ability of IUHCT to provide a platform on which postnatal allogeneic transplants, using the same-donor source that was used to perform the IUHCT, could be performed to increase the levels of donor cell engraftment or allow for successful organ transplant with minimal or no myeloablation or immunosuppression.*

*BM, bone marrow; TBI, total body irradiation.*

*In utero* hematopoietic cell transplantation may also induce donor specific tolerance and allow for postnatal solid organ transplants without the requirement for immunosuppressive conditioning (**Table 1**). Acceptance of donor skin grafts, a classic method of assessing donor specific tolerance, has been repeatedly demonstrated in the murine model of IUHCT with success dependent on the levels of donor cell chimerism (Chen et al., 2010). A renal transplant is potentially the most clinically relevant solid organ transplant in the setting of tolerance induction by IUHCT. Studies in the swine and canine model support the ability of donor specific tolerance induction by IUHCT to allow for successful postnatal same-donor renal transplants without immunosuppression. Interestingly, in the canine model, clinically insignificant but histologically detected mild chronic rejection of one recipient of a postnatal renal transplant following IUHCT was noted. This recipient had the lowest levels of peripheral blood donor cell chimerism (7%) at the time of renal transplant. The other renal transplant recipients had chimerism levels of 12–55% at the time of transplant and demonstrated no clinical or histologic evidence of rejection (Vrecenak et al., 2014). Donor cell engraftment levels of 7% are above what would be expected to induce donor cell tolerance and allow for successful non-myeloablative postnatal cellular transplants suggesting that chimerism levels that allow for successful postnatal solid organ transplants without immunosuppression may be different than those required for postnatal cellular transplants. Finally, tolerance induction by IUHCT to allow for xenogeneic solid organ transplants has also been investigated. Results from these limited studies highlight the potential of this approach to overcome the immune limitation to xenogeneic transplantation (Tanaka et al., 1998).

## CONCLUSION

*In utero* hematopoietic cell transplantation is a non-myeloablative non-immunosuppressive transplant approach that allows for donor cell engraftment and donor specific tolerance across immunologic barriers. It has the potential to treat a large number of congenital hematologic, genetic, and immunologic disorders which, because of advances in prenatal care, can be diagnosed before birth and before the maturation of the fetal immune system. Studies in murine and preclinical large animal models suggest that, in limited circumstances, a single IUHCT may result in high enough levels of donor cell engraftment to ameliorate the target disease. However, even in the absence of obtaining therapeutic levels of engraftment, the major benefit of IUHCT may be in the reliable induction of donor specific tolerance to allow for postnatal non-myeloablative same-donor cellular transplants to enhance engraftment to target levels with minimal treatment related toxicity. Although less clinically relevant at the current time, a similar approach of tolerance induction by IUHCT to allow for postnatal organ transplants without immunosuppression may hold promise in the future. In order to embrace the full potential of *in utero* tolerance induction for postnatal cellular and organ transplants, additional insights into the mechanisms involved in the induction and maintenance of tolerance including the role of peripheral regulatory cells as well as the barriers to engraftment that prevent the acquisition of donor specific tolerance in all recipients of IUHCT must be investigated.

## Conflict of Interest Statement

The author declares that the research was conducted in the absence of any commercial or financial relationships that could be construed as a potential conflict of interest.
